# Plant–plant interactions as a mechanism structuring plant diversity in a Mediterranean semi‐arid ecosystem

**DOI:** 10.1002/ece3.1770

**Published:** 2015-10-28

**Authors:** Antonio I. Arroyo, Yolanda Pueyo, Hugo Saiz, Concepción L. Alados

**Affiliations:** ^1^ Instituto Pirenaico de Ecología (CSIC) Av. Montañana 1005 P.O. Box 13.034 50080 Zaragoza Spain; ^2^ UMR 6553 Ecobio CNRS – University of Rennes 1 Av. du General Leclerc 35042 Rennes Cedex France

**Keywords:** Allelopathy, aridity, *Artemisia herba‐alba*, individual species–area relationship, interference, livestock grazing, Middle Ebro Valley, plant–plant interactions

## Abstract

Plant–plant interactions are among the fundamental processes that shape structure and functioning of arid and semi‐arid plant communities. Despite the large amount of studies that have assessed the relationship between plant–plant interactions (i.e., facilitation and competition) and diversity, often researchers forget a third kind of interaction, known as allelopathy. We examined the effect of plant–plant interactions of three dominant species: the perennial grass *Lygeum spartum*, the allelopathic dwarf shrub *Artemisia herba‐alba*, and the nurse shrub *Salsola vermiculata,* on plant diversity and species composition in a semi‐arid ecosystem in NE Spain. Specifically, we quantified the interaction outcome (IO) based on species co‐occurrence, we analyzed diversity by calculation of the individual species–area relationship (ISAR), and compositional changes by calculation of the Chao‐Jaccard similarity index. We found that *S. vermiculata* had more positive IO values than *L. spartum*, and *A. herba‐alba* had values between them. *Lygeum spartum* and *A. herba‐alba* acted as diversity repellers, whereas *S. vermiculata* acted as a diversity accumulator. As aridity increased, *A. herba‐alba* transitioned from diversity repeller to neutral and *S. vermiculata* transitioned from neutral to diversity accumulator, while *L. spartum* remained as diversity repeller. *Artemisia herba‐alba* had more perennial grass species in its local neighborhood than expected by the null model, suggesting some tolerance of this group to its “chemical neighbor”. Consequently, species that coexist with *A. herba‐alba* were very similar among different *A. herba‐alba* individuals. Our findings highlight the role of the nurse shrub *S. vermiculata* as ecosystem engineer, creating and maintaining patches of diversity, as well as the complex mechanism that an allelopathic plant may have on diversity and species assemblage. Further research is needed to determine the relative importance of allelopathy and competition in the overall interference of allelopathic plants.

## Introduction

The effect of biotic interactions on the structure and diversity of plant communities has been a central topic in ecology for the last half century. In classical ecological theories, only competitive interactions among plants have been considered to drive community structure and diversity (Grime 1973; Huston 1979). In communities from mid‐ to high‐productivity, a decrease in plant diversity was explained by increased competition and the exclusion of species with lower competitive capacity (Grime 1973). But, in the last years, many studies (Hacker and Gaines 1997; Bruno et al. 2003; Brooker et al. 2008; McIntire and Fajardo 2014) have recognized the key role of positive interactions driving diversity in plant communities. Facilitation is especially relevant in harsh environments (Callaway 2007; Soliveres and Maestre 2014), where the presence of nurse plants allows the persistence of stress‐intolerant species through expansion of their realized niches (Bruno et al. 2003). Therefore, positive interactions are crucial for increasing, maintaining, or preventing the loss of species diversity (Hacker and Gaines 1997; Michalet et al. 2006; Le Bagousse‐Pinguet et al. 2014), functional diversity (Schöb et al. 2012; Gross et al. 2013), and phylogenetic diversity (Valiente‐Banuet and Verdú 2007; Butterfield et al. 2013), not only at the local, but also at the regional and global scales (Cavieres et al. 2014).

In arid and semi‐arid communities, positive and negative interactions among plants occur simultaneously (Holzapfel and Mahall 1999). However, most studies that examine the net effect of plant–plant interactions usually forget that the interference that one plant can exert upon another goes beyond resources uptake (Holmgren et al. 1997; Holzapfel and Mahall 1999; Tielbörger and Kadmon 2000; Miriti 2006; but see Callaway et al. 1991). Allelopathy, the negative influence that a plant can have on the germination, growth, and survival of other plants due to release of toxic compounds called “allelochemicals” (Muller 1969; Chou and Waller 1983; Rice 1984), is a well‐known phenomenon in the fields of invasive plants (Callaway and Ridenour 2004) and agriculture (Chou 1999). But, research on allelopathic effects in natural ecosystems is still scarce, even though many plants may have potential allelopathic activity in the Mediterranean regions (Thompson 2005). Therefore, allelopathic species should also be considered in studies of plant–plant interactions in natural ecosystems.

Moreover, a recent study suggested that the mechanisms of allelopathic species structuring diversity in plant communities could be more complex than expected. Ehlers et al. (2014) found that the allelopathic Mediterranean species, *Thymus vulgaris* L., acts as diversity accumulator because it suppresses a superior competitor. In other words, allelopathy had positive net effects on diversity because of indirect facilitation (Brooker et al. 2008), in this mesic, Mediterranean, and species‐rich community. It remains unknown whether allelopathic species have similar effects on diversity in more arid areas, where competition for space is less important. In addition, in communities where allelopathic plants coexist with dozens of species, it has been observed that some plants may have resistance to allelopathic compounds (Vivanco et al. 2004; Grøndahl and Ehlers 2008; Thorpe et al. 2009). The extent of this resistance remains unclear, this is, whether plants have developed tolerance to *“*chemical neighbor*s”* because of co‐evolutionary adaptations or whether species that appear in the same community than an allelopathic plant simply avoid establishment nearby. Consequently, allelopathic species may have previously overlooked effects on the composition and diversity of plant communities.

The net outcome of plant–plant interactions changes as environmental stress changes (Bertness and Callaway 1994). At the global scale, exist a shift toward a more positive outcome in plant–plant interactions as stress level increases (He et al. 2013; Cavieres et al. 2014), although some research has noted a decline of positive interactions under extreme stress (Maestre et al. 2005; Smit et al. 2007; Michalet et al. 2014). In nature, several stressors do not occur separately, but they occur simultaneously and potentially interact. Although a growing body of studies have examined the effects of the interplay of several stress factors (*e.g.,* aridity and grazing) on biotic interactions (Maalouf et al. 2012; Mod et al. 2014; Verwijmeren et al. 2014), its effects on diversity, through regulation of biotic interactions, still remains unclear (Le Bagousse‐Pinguet et al. 2014). On the other hand, production of allelochemicals is greater in stressful environments in which there are harsh biotic and abiotic conditions such as water deficit, extreme temperatures, or physical damage from herbivory (Tang et al. 1995; Reigosa et al. 1999; Pedrol et al. 2006). Moreover, in stressed environments, plants may be more susceptible to allelochemicals (Reigosa et al. 1999; Pedrol et al. 2006) because they are already under stress. Therefore, when considering allelopathic species, the balance between facilitation and interference might change with increasing stress, leading to reduction or even prevention of positive net effects on diversity.

In this research, we aimed to evaluate the role of plant–plant interactions structuring diversity and species composition in a semi‐arid ecosystem NE Spain. Specifically, we assessed interaction outcome, diversity, and changes in species composition in the local neighborhood of the perennial grass *Lygeum spartum* L., the dwarf shrub *Artemisia herba‐alba* Asso., and the shrub *Salsola vermiculata* L. under different conditions of stress level (aridity and grazing). *Artemisia herba‐alba* and *S. vermiculata* are two species with similar physiognomy and plant traits. Both are long‐lived shrubs, with moderately dense canopy and deep roots, which are well‐known traits to host high plant diversity underneath in semi‐arid ecosystems (Callaway 2007; Parsons and Abrahams 2009; Pugnaire 2010). Therefore, they could potentially have the same facilitative effects on diversity. However, while *S. vermiculata* is considered an effective nurse plant, there are dozens of works in literature demonstrating the allelopathic nature of *A. herba‐alba*, dealing with ecological, physiological, biochemical, and medical approaches (Friedman et al. 1977; Escudero et al. 2000; Mohamed et al. 2010). On the other hand, *L. spartum* has very different plant traits from shrubs (dense and shallow rooting zone and very dense canopy) that confer to this species a high competitive ability for water and resources (Jurena and Archer 2003).

We expected mostly negative interactions between the allelopathic (*A. herba‐alba*) and competitive (*L. spartum*) plants and the other species present in this plant community, and mostly positive interactions between the nurse plant (*S. vermiculata*) and these other species. We hypothesized that species with mostly negative interaction would act as diversity repellers (i.e., would have a local neighborhood less diverse than expected) and that species with mostly positive interactions would act as diversity accumulators (i.e., would have a local neighborhood more diverse than expected) (sensu Wiegand et al. 2007). In particular, we expected that in drylands, where competition for space is not a dominant process, the allelopathic species would have a negative effect on diversity, in contrast to results found in mesic Mediterranean areas (Ehlers et al. 2014). Also, we hypothesized that if allelopathic compounds have determinant effects on germination and survival of other species present in community, only species adapted to allelochemicals will be able to coexist with the allelopathic species. Thus, we expected low compositional changes of species nearby the allelopathic species. Based on previous global observations, we expected a shift toward facilitation (or reduced competition) with increasing aridity. Also, we expected a similar shift with the presence of grazing pressure due to associational resistance (Olff and Ritchie 1998). Finally, we expected that allelopathy would partially or totally suppress the potential facilitative effects of the allelopathic species with increasing environmental stress (aridity and grazing).

## Materials and Methods

### Study area

**Figure 1:**
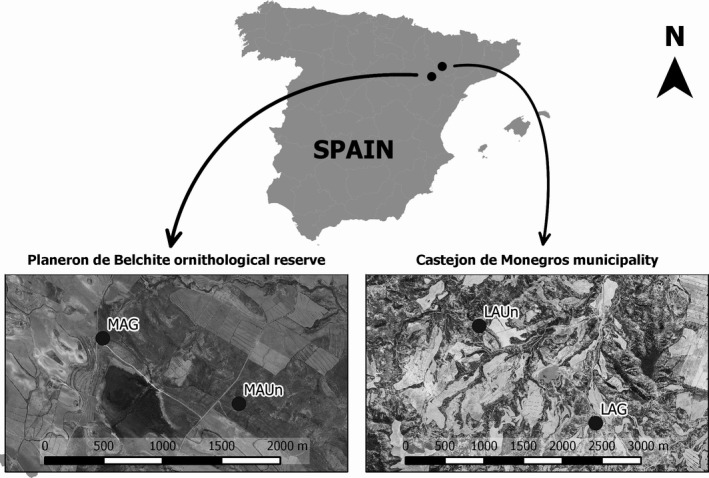
Locations of the four study sites in the Middle Ebro Valley of Spain. The distance between Planeron de Belchite ornithological reserve and Castejon de Monegros municipality is 53 km.

This study was conducted in the Middle Ebro Valley (NE Spain; Fig. 1). This region is one of the most arid areas in Spain, with an average annual temperature of 15°C and average annual precipitation of 353 mm year^−1^ (at 250 m.a.s.l., Zaragoza station, *n* = 50 years). The landscape mainly consists of flat‐bottomed valleys and low hills. Dry croplands and extensive sheep (*Rasa aragonesa*) production are the principal human activities (Pueyo 2005). The grass–shrub steppe community on noncultivated lands includes shrubs (*S. vermiculata*,* A. herba‐alba*, and *Suaeda vera* J.F.G.mel. among others), perennial grasses (*L. spartum*,* Brachypodium retusum* (Pers.) P.Beauv., *Dactylis glomerata* L.*,* and *Stipa parviflora* Desf. among others), and many annual and ephemeral herbs.

### Field surveys

We chose noncultivated lands in two zones. One zone is among the most arid regions in the Middle Ebro Valley (“El Planeron de Belchite” Ornithological Reserve, 41°22′24′′N, 00°37′55′′O) and has an average annual temperature of 15.4°C and average annual precipitation of 319 mm year^−1^; the other zone (Castejon de Monegros municipality, 41°41′36′′N, 00°09′49′′O) is less arid and has an average annual temperature of 14.7°C and average annual precipitation of 377 mm year^−1^ (Fig. 1; Table 1). The two zones have similar lithology, topography, and grazing regime (<0.7 head ha^−1^ year^−1^) (Pueyo et al. 2013). At each zone, we selected a grazed location and an ungrazed location for establishment of four study sites: LAUn (least arid, ungrazed), LAG (least arid, grazed), MAUn (most arid, ungrazed), and MAG (most arid, grazed) (Fig. 1; Table 1). LAG and MAG were continuously grazed until the date when sampling was carried out. In these communities, grazing tends to favor shrubs, while perennial grasses would be more dominant in the absence of grazing (Puigdefábregas and Mendizabal 1998). In the summer of 2010, we sampled six 250‐m transects at each study site (*n* = 24) using the point‐intercept method (Goodall 1952). Along each transect, we recorded the location of all species found each 20 cm. No distinction was made for ontogenetic stages.

**Table 1:** Location, grazing regime, climate (annual rainfall, mean annual temperature, and aridity index), and relative abundance (percent of species abundance respect to total species abundance, mean ± standard error) of target species (*Lygeum spartum*,* Artemisia herba‐alba* and *Salsola vermiculata*), and species abundance of different plant types (mean ± standard error) at each study site (see Pueyo et al. (2013) for more details about grazing management in the study sites). Annual rainfall and mean annual temperature data obtained from Digital Climatic Atlas of Aragón (http://anciles.aragon.es/AtlasClimatico/)

Study site	Location	Grazing	Annual rainfall (mm)	Mean annual temperature (°C)	Aridity index (°C mm^−1^)	Relative abundance of target species (%)	Plant type abundance (%)			
							Annuals	Perennial grasses	Dwarf shrubs	Shrubs
LAUn	Less arid	No	377	14.7	3.90	40.32 ± 1.29	41.93 ± 1.43	20.30 ± 0.70	24.61 ± 0.73	13.16 ± 1.55
LAG	Less arid	Yes	377	14.7	3.90	47.20 ± 3.46	52.51 ± 1.65	18.11 ± 0.81	21.97 ± 2.09	7.40 ± 0.91
MAUn	Most arid	No	319	15.4	4.83	54.64 ± 1.41	63.34 ± 0.78	13.36 ± 1.02	18.51 ± 0.63	4.79 ± 0.66
MAG	Most arid	Yes	319	15.4	4.83	38.05 ± 2.32	60.50 ± 2.94	15.79 ± 1.78	19.25 ± 1.31	4.46 ± 0.79

### Target species

We selected three perennial species that were present in all study sites to test our hypotheses: the competitive perennial grass *L. spartum*, the dwarf shrub and well‐known allelopathic *A. herba‐alba* (Friedman et al. 1977; Escudero et al. 2000; Mohamed et al. 2010), and the nurse shrub *S. vermiculata*. Together, these three target species accounted for a mean relative abundance of 45.35% ± 1.72%, ranging from a minimum of 38.05% ± 2.32% in MAG to a maximum of 54.64% ± 1.41% in MAUn (Table 1).

### Interaction outcome of target species

To determine whether each target species had mostly positive or negative interactions, we analyzed the spatial association of all possible pairs of species composed by target species and other species in the transect.

We assessed spatial association by comparing the number of co‐occurrences, *C*_r_, found for a given pair of species with the expected number of co‐occurrences, *C*_e_, that the pair of species would have based on their abundances. A co‐occurrence of a pair of species was considered when, in a transect, both species appeared together at the same point (Saiz and Alados 2012). Later, as co‐occurrences are count data, we compared *C*_r_ and *C*_e_ using a Poisson distribution with the *λ* parameter fitted to *C*_e_. When *C*_r_ was significantly greater than *C*_e_ for a pair of species, this means that they co‐occurred more times than expected by chance, and therefore, we assumed a positive spatial association. On the other hand, when *C*_r_ was significantly less than *C*_e_ for a pair of species, this means that they co‐occurred less often than expected by chance, and we assumed a negative association. Non‐significant differences between *C*_r_ and *C*_e_ indicate a random spatial association. Although co‐occurrence of species can be driven not only by plant‐plant interactions, but also by, for example, similar habitat requirements, co‐occurrence is generally accepted as an indicator of plant interactions in drylands (Saiz and Alados 2012; Soliveres et al. 2014). Thus, we interpreted positive and negative associations as proxies for positive and negative interactions. Then, we computed the number of positive and negative associations (i.e., positive and negative interactions) for each target species, and the interaction outcome (IO) of each target species based on its number of positive and negative interactions with the other plants in the community:

$$\begin{array}{cc} \text{Interaction Outcome (IO)}\\ & =\frac{\text{Positive interactions}-\text{Negative interactions}}{\text{Total interactions}} \end{array}$$

For each target species, an IO value of 1 indicates positive interactions with all species along the transect, an IO value of −1 indicates negative interactions with all species, and an IO value of 0 indicates the same number of positive and negative interactions. Differences in IO among target species and sites were analyzed with two‐way ANOVA. Tukey′s post hoc honest significant difference (HSD) tests were used to detect differences for pairs of target species and sites. Assessment of spatial associations and statistical analyses were performed with R (R Core Team 2013).

### Diversity patterns near target species

We analyzed the diversity in the local neighborhood of individuals of the three target species. For this purpose, we followed the ISAR (individual species–area relationships) method proposed by Wiegand et al. (2007). The ISAR_(*d*)_ can be defined as the expected number of species within a distance *d* of a given individual of the target species *t,*

$${\text{ISAR}}_{\left(d\right)}=\sum_{j=1}^{S}\left[1-P_{t,j}\left(0,d\right)\right]$$

where *P*_*t*,* j*_(0, *d*) is the probability that species *j* was not present within distance *d* of individuals of target species *t*. Thus, the ISAR_(*d*)_ value will be the sum of 1 − *P*_*t*,* j*_(0, *d*) for all species *j* present in the transect (Wiegand et al. 2007). ISAR_(*d*)_ was calculated along 1500‐m transect (six 250‐m transects) at each study site to a maximal distance of 4 m (*d *=* *4), which is considered sufficient for detection of plant–plant interactions (Rayburn and Wiegand 2012). The ISAR method considers plant–plant interactions at several scales, disentangling the spatial dependency of the interactions. A predominance of positive interactions would lead to diversity accumulation in the local neighborhood of individuals of the target species; conversely, a predominance of negative interactions would lead to diversity repulsion. On the other hand, a neutral balance of positive and negative interactions or the presence of only weak interactions with other species would lead to a local neighborhood as diverse as expected.

We calculated a confidence envelope using a Monte Carlo test with 199 heterogeneous Poisson null model simulations (Wiegand and Moloney 2004) to determine whether for a given distance *d*, the ISAR_(*d*)_ of a target species was significantly greater or less than expected by chance (Wiegand et al. 2007; Rayburn and Wiegand 2012). Each heterogeneous Poisson null model simulation replaces individuals of the target species randomly within the maximal distance in which plant–plant interactions are expected to occur. Hence, for distances greater than 4 m (*d *=* *4), the spatial distribution of target species individuals was maintained, and for distances less than 4 m, the spatial structure was removed. If the ISAR_(*d*)_ was greater than the fifth largest value (or less than the fifth lowest value) from simulations, then individuals of that target species were surrounded at distance *d* by more (or fewer) species than expected by the null model, with *α *≈ 0.05. If the ISAR_(*d*)_ was within the confidence envelope, then individuals of that target species were surrounded by the same number of species than expected by the null model. We assessed the significance of the relationship between interaction outcome (IO) of target species and ISAR values at local neighborhood (*d *=* *20 cm) with a linear model.

The local neighborhood of individuals of a target species could be as diverse as expected by the null model even when such target species interacts significantly with a few species or plant types. To assess this effect, we classified plant species into four types based on life form: annual, perennial grass, shrub, and small shrub. Then, we computed ISAR_(*d*)_ for each combination of target species and plant type. Thus, at each study site, we performed ISAR analyses by considering the entire diversity (excluding *j* when *j *= *t*) and by considering different plant types separately.

ISAR analyses and comparisons with null models were performed with MATLAB R2010b. The linear relationship for IO and ISAR_(*d *= 20)_ was determined with R (R Core Team 2013).

### Compositional changes of species associated with target species

In order to assess compositional changes of species spatially associated with target species, we employed the Chao‐Jaccard similarity index (Chao et al. 2005). For each target species, we calculated this index for all possible pairs of transects of the same study site, using data of co‐occurrence between pair of species (number of times that target species and other species appeared at the same point in a transect). Chao‐Jaccard index is based on the probability that two randomly chosen individuals that co‐occur with the target species (one from each of two transects) both belong to any of the species shared by the two transects (Chao et al. 2005) (i.e., species that co‐occur with the target species in both transects). This index considers the similarity of the species list that co‐occurs with a given target species, and the similarity of the relative abundances of co‐occurring species. Thus, a high similarity among transects indicates that nearly the same species co‐occur with a target species and approximately the same frequencies. The Chao‐Jaccard similarity index was calculated using the vegan package in R (Oksanen et al. 2013). We compared Chao‐Jaccard similarity index among target species at each study site by use of one‐way ANOVA. Tukey′s post hoc HSD test was employed to detect differences among pairs of target species. These statistical analyses were performed with R (R Core Team 2013).

## Results

### Outcome of plant–plant interactions of target species

**Figure 2:**
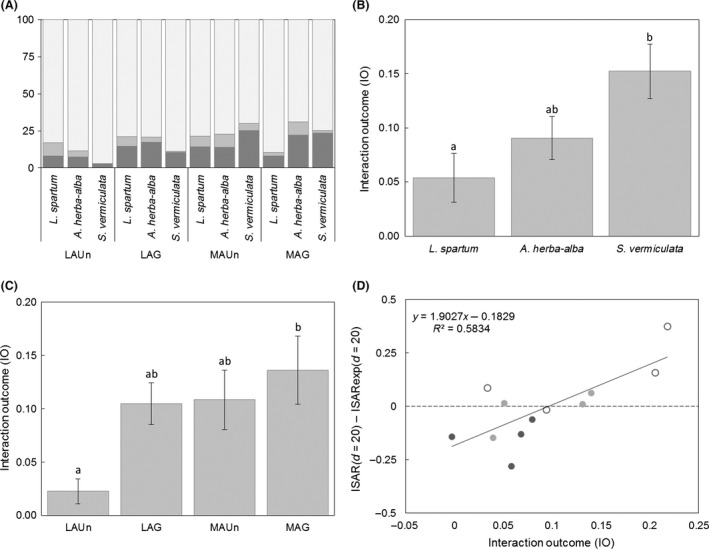
(A) Percentage of positive (dark gray), negative (medium gray) and neutral (light gray) interactions of target species at each study site. (B) Mean interaction outcome (IO) values (bars) and standard errors (error bars) of *Lygeum spartum* (*n* = 21), *Artemisia herba‐alba* (*n* = 24) and *Salsola vermiculata* (*n* = 21). Different letters indicate significant differences between target species. (C) Mean IO values (bars) and standard errors (error bars) at LAUn (*n* = 16), LAG (*n* = 18), MAUn (*n* = 18) and MAG (*n* = 18). Different letters indicate significant differences. (D) Relationship between IO and species diversity within 20 cm (ISAR_(*d = 20*)_ – ISAR_exp(*d = 20*)_) of target species individuals at different study sites (*R* ^2^ = 0.583, *P *=* *0.004). *Lygeum spartum*: dark gray dots; *Artemisia herba‐alba*: light gray dots; and *Salsola vermiculata*: open circles.

Positive and negative interactions were found for all three target species (*L. spartum*,* S. vermiculata* and *A. herba‐alba*) (Fig. 2A). In particular, these target species had significantly positive and negative interactions with approximately 20% of the plant species in the community (Fig. 2A). Overall, the three target species had positive IO values, but there were significant differences among the different species (*F*_2,54_ = 5.48, *P *=* *0.007). Tukey′s post hoc HSD test showed that *S. vermiculata* had a significantly more positive IO value than *L. spartum*, and that *A. herba‐alba* had an intermediate IO value between them (Fig. 2B).

The IO differed significantly among the study sites (*F*_3,54_ = 3.65, *P *=* *0.018). In particular, Tukey′s post hoc HSD test showed that the IO was significantly more positive at MAG than at LAUn and that the IO had intermediate values at LAG and MAUn, (Fig. 2C). On the other hand, the interaction between target species and study site was non‐significant (*F*_6,54_ = 1.38, *P *=* *0.238), indicating that the differences found in IO among the target species were similar at all study sites.

### Diversity patterns of nearby target species

The IO of target species and the diversity near individuals of target species had a significantly positive relationship (*F*_1,10_ = 13.93, *P *=* *0.004; Fig. 2D). In other words, a predominance of positive interactions was associated with diversity accumulation and a less positive balance was associated with diversity repulsion.

**Figure 3:**
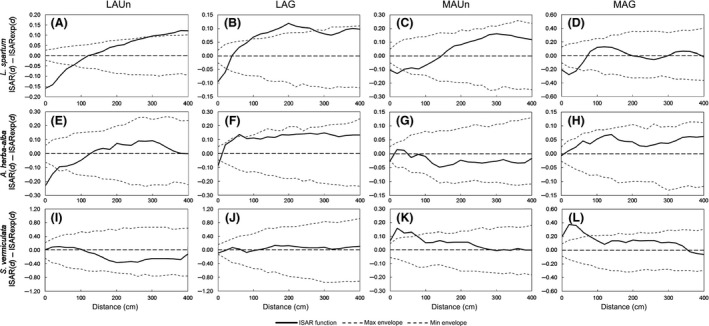
Individual species–area relationship (ISAR) curves of target species at each study site. Solid lines are ISAR values (ISAR_(*d*)_ – ISAR_exp(*d*)_, where ISAR_exp(*d*)_ is the average of 199 heterogeneous Poisson null model simulations), and dotted gray lines are the 95% confidence envelope (fifth largest and the fifth smallest values from the simulations) for each spatial scale. When ISAR was above the confidence envelope, the local neighborhood is more diverse than expected by the null model (diversity accumulation). When ISAR was below the confidence envelope, the local neighborhood is less diverse than expected by the null model (diversity repulsion). When ISAR was within the confidence envelope, the local neighborhood was as diverse as expected by the null model.

ISAR analysis showed that the perennial grass *L. *s*partum* acted as diversity repeller in all study sites at distances less than 100 cm (Fig 3A–D); there were significant and positive departures of the ISAR curve in the less arid study sites at greater distances (Fig 3A and B). *Lygeum spartum* mainly repelled other perennial grasses (Table 2; see Fig. S1 in Supporting Information), and it had greater diversity of nearby annuals and dwarf shrubs species than expected by the null model (Table 2; Fig. S1).

**Table 2:** Summary of ISAR curves for *Lygeum spartum*,* Artemisia herba‐alba* and *Salsola vemiculata* based on plant type at each study site (see Figs S1–S3)

	LAUn	LAG	MAUn	MAG
*L. spartum*				
Annuals	(−), + +	+ +	0	0
Perennial grasses	− −, (+)	− −	− −	−
Dwarf shrubs	(−), +	(−), + +	− −	− −
Shrubs	− −	− −	(−)	0
*A. herba‐alba*				
Annuals	(−)	0	0	(+)
Perennial grasses	(−), + +	+	+ +	+
Dwarf shrubs	−	−	0	− −
Shrubs	− −	−	− −	−
*S. vermiculata*				
Annuals	(−)	0	+ +	+ +
Perennial grasses	0	0	+	(+)
Dwarf shrubs	0	0	−	(−)
Shrubs	0	0		

+, presence of diversity accumulation at some distance; −, presence of diversity repulsion at some distance; and 0, no departure from the confidence envelope at any distance. Symbols separated by commas indicate different behaviors at different scales; ++ and − − indicate large departures from confidence envelope; and symbols within parentheses indicate marginal departures from confidence envelope.

The allelopathic species *A. herba‐alba* acted as diversity repeller in the less arid study sites (LAUn and LAG; Fig 3E and F); there were significant departures of the ISAR curve at distances less than 50 cm in LAUn (Fig. 3E) and at distances close to 0 cm in LAG (Fig. 3F). On the other hand, *A. herba‐alba* individuals had a local neighborhood as diverse as expected by the null model in the most arid study sites (MAUn and MAG; Fig 3G and H). Calculation of ISAR for different plant types showed that *A. herba‐alba* repelled other shrubs and dwarf shrubs species (Table 2; Fig. S2) and, although facilitative effects on the whole plant diversity were not found, this species acted as diversity accumulator of perennial grasses in all study sites, as it had more species of perennial grasses in the local neighborhood of its individuals than expected by the null model (Table 2; Fig. S2).

The shrub *S. vermiculata* was neutral in the less arid study sites (LAUn and LAG; Fig 3I and J), but acted as a diversity accumulator in the most arid sites (MAUn and MAG; Fig 3K and L) at distances of 0–80 cm. This effect was mostly for annuals and perennial grass species (Table 2; Fig. S3).

### Compositional changes of species associated with target species

**Figure 4:**
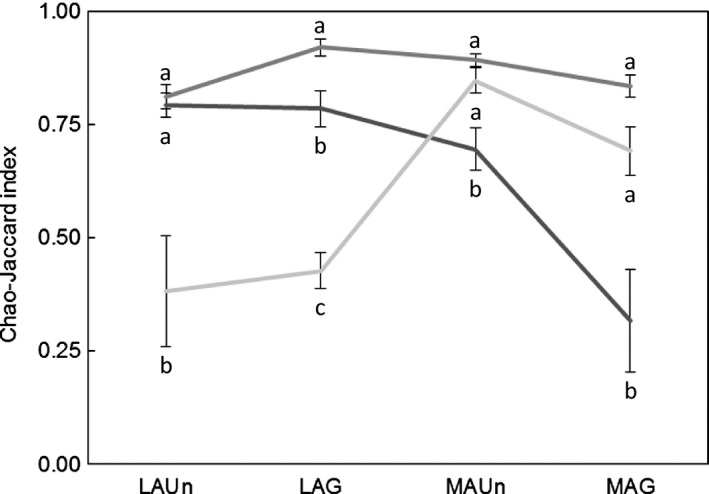
Means (line) and standard errors (error bars) of the Chao‐Jaccard similarity index of *Lygeum spartum* (n_LAU__n_ = 15, n_LAG_ = 15, n_MAU__n_ = 10, n_MAG_ = 6; dark gray), *Artemisia herba‐alba* (n_LAU__n_ = 15, n_LAG_ = 15, n_MAU__n_ = 15, n_MAG_ = 15; medium gray) and *Salsola vermiculata* (n_LAU__n_ = 3, n_LAG_ = 15, n_MAU__n_ = 15, n_MAG_ = 15; light gray), where n_site_ is the number of transects comparisons per study site. The number of comparisons differ because only transects in which the target species were present were analyzed. Different letters indicate significant differences between target species.

There were significant differences in the Chao‐Jaccard similarity index among target species at all study sites (*F*_2,30_ = 18.88, *P *<* *0.001 for LAUn; *F*_2,42_ = 55.57, *P *<* *0.001 for LAG; *F*_2,37_ = 10.96, *P *<* *0.001 for MAUn; *F*_2,33_ = 17.21, *P *<* *0.001 for MAG; Fig. 4). Tukey′s post hoc HSD test showed that *L. spartum* had significantly lower similarity than *A. herba‐alba* and *S. vermiculata* in the most arid study sites (MAUn and MAG), but there were non‐significant differences in the Chao‐Jaccard similarity index between *A. herba‐alba* and *S. vermiculata* in those study sites (Fig. 4). On the other hand, in the less arid study sites (LAUn and LAG), the diversity associated with *S. vermiculata* had a significantly lower similarity between individuals of this species than diversity associated with *L. spartum* and *A. herba‐alba*. There were no differences in the Chao‐Jaccard similarity index between *L. spartum* and *A. herba‐alba* at LAUn, but *L. spartum* had a significantly lower similarity than *A. herba‐alba* at LAG (Fig. 4).

## Discussion

The purpose of this study was to assess the effects of plant–plant interactions, and its modulation along contrasting conditions of aridity and grazing pressure, of three dominant species on plant diversity and species composition in a semi‐arid ecosystem. Our results indicated that *S. vermiculata* (nurse plant) had in its local neighborhood more species than expected by the null model, while *L. spartum* (perennial grass) and *A. herba‐alba* (allelopathic plant) had fewer species than expected by the null model. Overall, perennial grasses tolerated allelopathic compounds of *A. herba‐alba*. Moreover, the composition of species that appeared near *A. herba‐alba* was very similar among transects. Together, these results suggested that allelopathic activity of *A. herba‐alba* may act as a biotic filter in species assemblage. The role of *S. vermiculata* and *A. herba‐alba* on diversity changed as stress level increased, but *L. spartum* exhibited no such change. Aridity rather than grazing seemed to be the main driver of those changes.

The nurse shrub *S. vermiculata* had a significantly more positive net interaction outcome than the perennial grass *L. spartum*, while the allelopathic dwarf shrub had a net interaction outcome between them. In addition, individuals of *S. vermiculata* had more species around them than expected only by chance. In general, shrubs are considered good acting as nurse plants (Gómez‐Aparicio 2009) in a wide variety of ecosystems. The greater number of species and plant density under the canopy of *S. vermiculata* than under *A. herba‐alba* and *L. spartum* could be due to its nursing effect during plant establishment. *Salsola vemiculata* greatly improves the abiotic conditions under its canopy, such as water infiltration and nutrient enrichment, and thus improves the survival and growth of seedlings. More specifically, *S. vermiculata* facilitated annuals and also perennial grasses. Numerous studies have documented positive interactions between shrubs and annuals in drylands throughout the world (Wilby and Shachak 2004; Holzapfel et al. 2006; López et al. 2009).

The perennial grass *L. spartum* repelled diversity of all plant types at short distances. Perennial grasses have a dense root system in the upper soil layers (Jackson et al. 1996) which exerts a strong belowground competition (Armas and Pugnaire 2011), mainly in water uptake. It has been argued that competition for resources such as water is especially important during the establishment of seedlings (Jankju 2013). A higher efficiency of *L. spartum* in water uptake would increase the mortality of nearby seedlings, thus explaining its strong role as diversity repeller. The shrub *S. vermiculata* facilitated perennial grasses, but *L. spartum* did not facilitate shrubs. This finding is in line with studies that identified an ontogenetic shift in the relationship between shrubs and grasses; shrubs facilitate grasses at early stages of development, and established grasses finally replace and impede establishment of new shrubs (Armas and Pugnaire 2005).

Surprisingly, in the less arid study sites, *L. spartum* had more species of annuals and dwarf shrubs at medium and large distances than expected by the null model. We suggest two possible alternative explanations for this observation. First, the most of species of these plant types may appear at the edge of *L. spartum* individuals because competitive exclusion is less intense than within *L. spartum* clumps and stress is not as high as in open bare soil (Armas and Pugnaire 2011; Pescador et al. 2014). Second, it seems that the aggregated spatial pattern at large scales may reflect a heterogeneous spatial pattern in the environment (Wiegand and Moloney 2014). In other words, perennial grasses, annuals, and dwarf shrubs may have similar environmental requirements heterogeneously distributed along transects.

Ehlers et al. (2014) provided evidence that allelopathic plants can have potential positive effects on plant diversity in natural ecosystems. However, the allelopathic species *A. herba‐alba* had no positive net effects on diversity. It may be that in plant communities of arid and semi‐arid environments, where facilitation is a dominant process (Soliveres and Maestre 2014), allelopathy may constitute an evolutionary mechanism to avoid becoming nurse species (Van der Putten 2009) and thus reduce the negative impact that beneficiaries may cause on nurses fitness because of resource competition (Holzapfel and Mahall 1999; Schöb et al. 2014). Nevertheless, we found higher diversity of perennial grasses than expected in the local neighborhood of *A. herba‐alba*. Interestingly, facilitation of perennial grasses by *A. herba‐alba* occurred at all study sites and levels of stress. Previous research indicated that some species that coexist with allelopathic species may exhibit a tolerance to its “chemical neighbor” (Grøndahl and Ehlers 2008) due to co‐evolution. In agreement, *A. herba‐alba* generally had higher similarity in its understory plant composition than the competitive (*L. spartum*) and the nurse (*S. vermiculata*) species. This means that abundance and identity of species associated with *A. herba‐alba* were more similar among *A. herba‐alba* than the abundance and identity of species associated with *L. spartum* and *S. vermiculata* among themselves. It is possible that, beyond diversity repulsion, the allelopathic activity of *A. herba‐alba* acts as an environmental filter that reduces the species pool that can occur nearby, because only species adapted to its allelochemicals (*i.e.,* perennial grasses) can coexist with *A. herba‐alba*. Thus, we could observe two plant strategies to coexist with *A. herba‐alba* in the same community: tolerance to allelopathic compounds, as in the case of perennial grasses, and avoidance of direct coexistence under the *A. herba‐alba* canopy, in the case of other plant types. These two markedly different strategies – tolerance and avoidance – have different underlying mechanisms, and there is a clear need to be further examined to better understand plant interactions and predict community dynamics during conservation and restoration practices.

Despite *A. herba‐alba* and *L. spartum* being diversity repellers, all three target species had a positive net interaction outcome. These apparently contradictory results could be explained by one particularity in the method used to assess species association at the pairwise level (Saiz and Alados 2012). For those pair of species in which the abundance of one of the species was very low, we were not able to detect significant negative spatial associations. In particular, we were not able to detect a negative association when, in a transect, the number of expected co‐occurrences, *C*_e_, was lower than approximately *C*_e_ = 2.9, because the minimum possible number of co‐occurrences, *C*_r_, for the pair of species (*C*_r_ = 0, species do not co‐occur in the field) is included within the 95% confidence interval (Saiz and Alados 2012), and a neutral association was assigned in those cases. Therefore, as we could only detect positive or neutral associations between target species and rare species, the net IO of target species could be biased toward positive values. This limitation could explain why *L. spartum* and *A. herba‐alba*, which were expected to have negative IO values (i.e., a predominance of negative interactions), exhibited a positive net IO (i.e., a predominance of positive interactions). Nevertheless, these two species had smaller IO values than *S. vermiculata*, suggesting that, actually, *A. herba‐alba* and *L. spartum* were less facilitative species.

In line with predictions of the *Stress‐Gradient Hypothesis* (SGH), we found more positive IO values with increasing stress level (Bertness and Callaway 1994; He et al. 2013). Interestingly, significant differences in the IO occurred only between the reference site (LAUn) and the site with the greatest stress (MAG). This suggests that, although each stressor alone has little effect, the combined effects of multiple stressors drive the net interaction outcome (Le Bagousse‐Pinguet et al. 2014). This reinforces the view that the severity of different environmental stressors (*e.g.,* aridity in drylands) must be considered when interpreting the different effects that a species has on diversity (Mod et al. 2014).

In accordance with previous works that found amelioration of abiotic stress was more important than grazing protection (Gómez‐Aparicio 2008; Howard et al. 2012), our results indicated that aridity rather than livestock grazing was the main factor modulating the role of the target species on diversity. In our study, higher aridity caused both an increase in facilitation (from neutral to diversity accumulator) and a decrease in interference (from diversity repeller to neutral) in the local neighborhood of the nurse shrub *S. vermiculata* and the allelopathic dwarf shrub *A. herba‐alba*, respectively. As expected, we found that allelopathy of *A. herba‐alba* seems to counterbalance its potential facilitative effects on diversity (i.e., species accumulation) nearby its individuals (Jankju 2013) at most arid sites. Thus, when allelopathic species are involved, the balance between interference and facilitation may depend of the number of species able to tolerate allelopathic compounds, because negative effects on intolerant species can be counteracted by positive effects on tolerant species. On the other hand, aridity is an important driver of interactions when woody nurses are involved, but this does not hold for perennial grasses (Soliveres et al. 2014). Our finding that aridity did not modulate the negative effect of *L. spartum* on diversity agrees with this finding. Together, these results suggest species‐specific traits may influence biotic interactions more than or as much as environmental stress (Maalouf et al. 2012; Mod et al. 2014; Soliveres et al. 2014).

There are contrasting results in the literature regarding the effect of grazing pressure. Some research indicates that greater grazing pressure leads to positive plant–plant interactions due to grazing protection (Graff et al. 2007; Smit et al. 2007), especially when there are other stressor such as water scarcity (Anthelme and Michalet 2009; Soliveres et al. 2012). However, other research of areas with low productivity indicated that the effect of grazing driving positive interactions is less important than other factors such as environmental conditions (Smit et al. 2009; Howard et al. 2012). Our results are more in line with these later studies, because we found that livestock grazing alone was not enough to change the role of the target species on diversity. We suggest two possible explanations for our results. First, livestock grazing pressure in the study area was at a sustainable level (Pueyo 2005) and may be too low to have any effects on biotic interactions and diversity. Second, our ungrazed study sites were not totally free of grazing by wild animals such as rabbits (*personal observation*), and this would reduce differences between ungrazed and grazed sites.

In conclusion, our results point out the major role that biotic interactions of dominant species have in shaping the structure of a plant community (Le Roux et al. 2014). Specifically, given the strong implications of vegetation patches in the function of arid and semi‐arid ecosystems (Aguiar and Sala 1999), our results highlight the importance of nurse shrubs as ecosystem engineers, as they created and maintained vegetation patches with high diversity in our study area. On the other hand, the allelopathic species had mainly a negative effect on diversity, contrary to the situation in mesic Mediterranean ecosystems. Interestingly, other species appeared to develop two different strategies to coexist with the allelopathic species *A. herba‐alba*: tolerance, as exemplified by perennial grasses, and avoidance, as exemplified by the other plant types. Usually, studies involving allelopathic species mainly focus on assessing, in greenhouse experiments, the negative impact of isolated compounds or extracts of fresh material on seed germination and plant growth (Escudero et al. 2000; Gómez‐Aparicio and Canham 2008; Tilaki et al. 2013). Such studies often employ model species that do not coexist with the allelopathic plant in nature (*e.g., Lactuca sativa,* lettuce; Escudero et al. 2000; Jankju 2013). Hence, the ecological consequences of allelopathic species structuring plant diversity in natural communities are poorly understood (Chou 1999). Our results provide valuable information about the role of an allelopathic dwarf shrub on plant diversity and species assemblage at local scale in natural ecosystems, although these conclusions should be taken with caution as they are based on a single allelopathic species. Further field experiments will be necessary to determine the relative importance of allelopathy and competition in the overall interference of allelopathic plants (Nilsson 1994; Inderjit and Callaway 2003). Also, further research should test the generality of these findings on allelopathic species of other semi‐arid communities, and the variability in chemical composition among individuals and genotypes.

## Data Accessibility

All data are available from the Dryad Digital Repository: http://dx.doi.org/10.5061/dryad.q1r50.

## Conflict of Interest

None declared.

## Supporting information


**Figure S1.** Individual species–area relationship (ISAR) curves of *Lygeum spartum* according to plant type at each study site. Here and below: these curves can be interpreted as described in the legend of Fig. 3; Table 2 provides summaries of all curves.

**Figure S2.** Individual species–area relationship (ISAR) curves of *Artemisia herba‐alba* according to plant type at each study site.

**Figure S3.** Individual species–area relationship (ISAR) curves of *Salsola vermiculata* according to plant type at each study site.
