# Supplementary figures and images for: Plant–plant interactions as a mechanism structuring plant diversity in a Mediterranean semi‐arid ecosystem
Source: Ecol Evol. 2015 Oct 28;5(22):5305–17. doi: 10.1002/ece3.1770 (PMC6102514; doi:10.1002/ece3.1770)

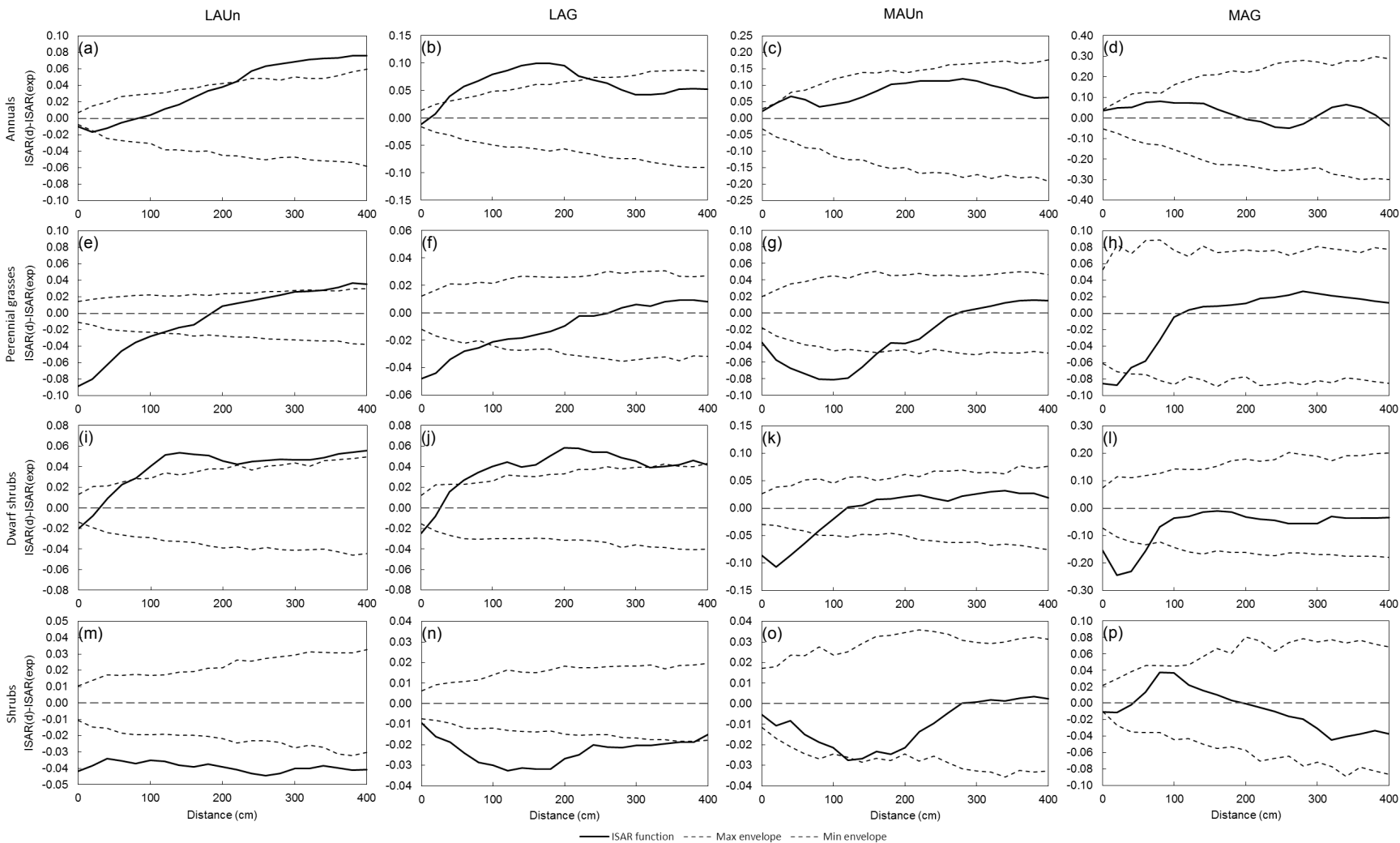

Supplement: Supplementary file 1 — Figure S1. Individual species–area relationship (ISAR) curves of Lygeum spartum according to plant type at each study site. Here and below: these curves can be interpreted as described in the legend of Fig. 3; Table 2 provides summaries of all curves. [file ECE3-5-5305-s001.pdf]

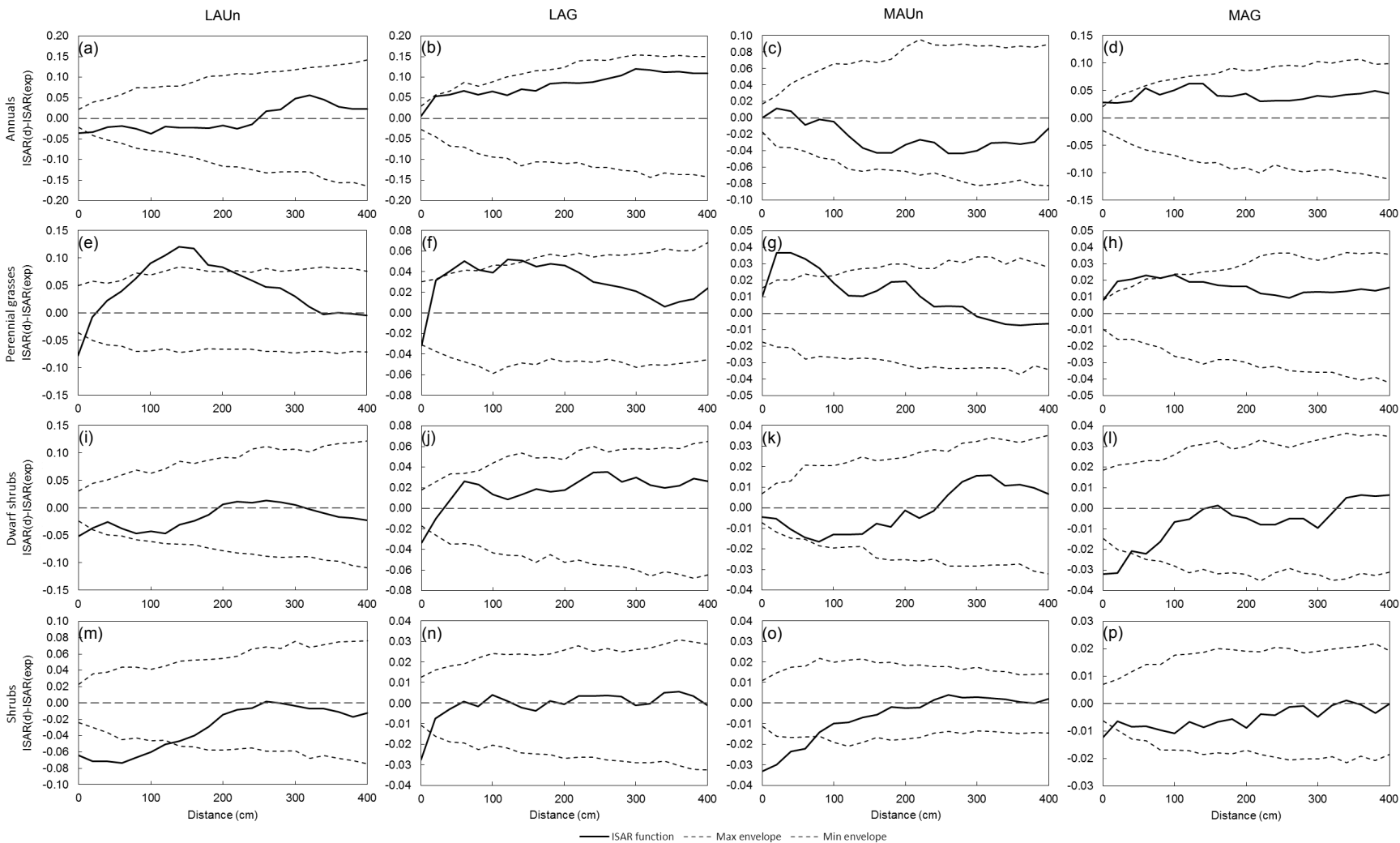

Supplement: Supplementary file 2 — Figure S2. Individual species–area relationship (ISAR) curves of Artemisia herba‐alba according to plant type at each study site. [file ECE3-5-5305-s002.pdf]

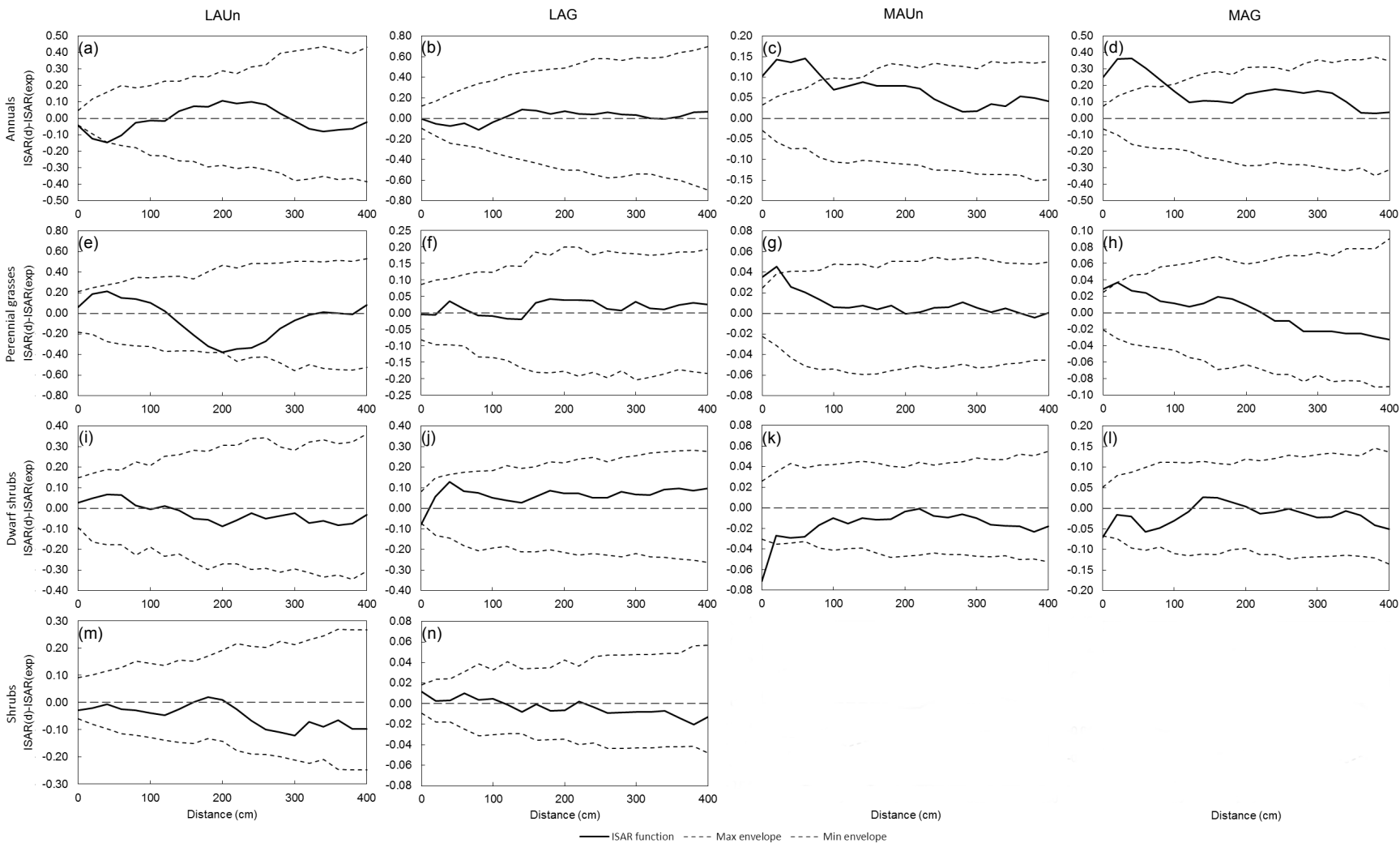

Supplement: Supplementary file 3 — Figure S3. Individual species–area relationship (ISAR) curves of Salsola vermiculata according to plant type at each study site. [file ECE3-5-5305-s003.pdf]
